# A Novel Gold Film-Coated V-Shape Dual-Core Photonic Crystal Fiber Polarization Beam Splitter Covering the E + S + C + L + U Band

**DOI:** 10.3390/s21020496

**Published:** 2021-01-12

**Authors:** Yuwei Qu, Jinhui Yuan, Shi Qiu, Xian Zhou, Feng Li, Binbin Yan, Qiang Wu, Kuiru Wang, Xinzhu Sang, Keping Long, Chongxiu Yu

**Affiliations:** 1 State Key Laboratory of Information Photonics and Optical Communications, Beijing University of Posts and Telecommunications, Beijing 100876, China; laigen007@163.com (Y.Q.); qiushi@bupt.edu.cn (S.Q.); yanbinbin@bupt.edu.cn (B.Y.); krwang@bupt.edu.cn (K.W.); xzsang@bupt.edu.cn (X.S.); cxyu@bupt.edu.cn (C.Y.); 2 Research Center for Convergence Networks and Ubiquitous Services, University of Science & Technology Beijing (USTB), Beijing 100083, China; zhouxian219@ustb.edu.cn (X.Z.); longkeping@ustb.edu.cn (K.L.); 3 Photonics Research Centre, Department of Electronic and Information Engineering, The Hong Kong Polytechnic University, Hung Hom, Hong Kong; enlf@polyu.edu.hk; 4 Department of Physics and Electrical Engineering, Northumbria University, Newcastle upon Tyne NE1 8ST, UK; qiang.wu@northumbria.ac.uk; 5 Key Laboratory of Nondestructive Test (Ministry of Education), Nanchang Hangkong University, Nanchang 330063, China

**Keywords:** V-shape dual-core photonic crystal fiber, polarization beam splitter, surface plasmon resonance effect, extinction ratio, insertion loss

## Abstract

In this paper, a novel gold film-coated V-shape dual-core photonic crystal fiber (V-DC-PCF) polarization beam splitter (PBS) based on surface plasmon resonance effect is proposed. The coupling lengths of the X-polarization (X-pol) and Y-polarization (Y-pol) and the corresponding coupling length ratio of the proposed V-DC-PCF PBS without gold film and with gold film are compared. The fiber structure parameters and thickness of the gold film are optimized through investigating their effects on the coupling lengths and coupling length ratio. As the propagation length increases, the normalized output powers of the X-pol and Y-pol of the proposed V-DC-PCF PBS at the three wavelengths 1.610, 1.631, and 1.650 μm are demonstrated. The relationships between the extinction ratio (ER), insertion loss (IL) and wavelength for the three splitting lengths (SLs) 188, 185, and 182 μm are investigated. Finally, it is demonstrated that for the proposed V-DC-PCF PBS, the optimal SL is 188 μm, the ILs of the X-pol and Y-pol are less than 0.22 dB, and the splitting bandwidth (SB) can cover the E + S + C + L + U band. The proposed V-DC-PCF PBS has the ultra-short SL, ultra-wide SB, and ultra-low IL, so it is expected to have important applications in the laser, sensing, and dense wavelength division multiplexing systems.

## 1. Introduction

Since the first photonic crystal fiber (PCF) was fabricated by Russell et al. in 1996 [1], PCFs have been investigated extensively and applied in different optical fields, such as fiber laser, sensing, optical communication, and so on [2,3,4,5,6,7,8]. At present, the PCF-based optical devices, including polarization beam splitter (PBS), polarization filter, modulator, etc., have become the indispensable components in the all-fiber optical systems [9,10,11,12,13,14].

In recent years, the dual-core PCF (DC-PCF) PBS based on the coupled mode theory has been widely investigated [15,16,17,18,19]. In 2016, Zi et al. reported a simple DC-PCF PBS, whose splitting lengths (SLs) are 249 and 506 μm and splitting bandwidths (SBs) are 17 and 12 nm at wavelengths 1.55 and 1.31 μm, respectively [20]. In the same year, Wang et al. proposed a liquid crystal-filled DC-PCF PBS, whose SL is 890.5 μm and SB covers the S + C + L band [21]. In 2017, He et al. designed an octagonal lattice DC-PCF PBS with the five elliptical air holes, whose SL is 105 μm and SB covers the S + C + L band [22]. In 2017, Wang et al. demonstrated a surface plasmon resonance (SPR) effect-based DC-PCF PBS filled with elliptical gold wire, whose SL is 1.079 mm and SB only covers the C band [23]. In 2018, Wang et al. achieved a short DC-PCF PBS with the liquid filled in the central and two elliptical air holes, whose SL is 78 μm and SB only covers the C band [24]. In 2019, Lou et al. investigated an ultrashort SPR effect-based DC-PCF PBS coated with gold film, whose SL is only 47.26 μm and SB cannot cover the C band [25]. From the previous works, the performances of the DC-PCF PBS can be obviously improved by introducing the elliptical air holes, changing the lattice arrangement of air holes, and selectively filling or coating the air holes with the liquid crystal, liquid, metal wire, and metal film.

Up to now, the fabrication technology of the PCFs with the circular air holes arranged in a hexagonal lattice has been developed more mature [26,27,28,29,30,31,32]. Many fabrication methods have been reported, including stack-and-draw, 3D printing, femtosecond laser drilling, etc. [33,34,35,36,37,38,39]. In contrast, it is more difficult to fabricate the PCFs when the air holes are arranged in the rectangle and octagonal shapes or the elliptical air holes exist. Especially when selectively filling the metal wire or coating the metal film into the air holes of the PCFs, the difficulty of fabrication is further increased. Since Sazio et al. and Russell et al. Firstly demonstrated the gold film-coated PCF in 2006 [40] and gold wire-filled PCF in 2008 [41], respectively, there are some reports on fabricating the gold film-coated and gold wire-filled PCFs by different methods [42,43,44,45,46,47,48]. At present, it is becoming a research hotspot to design and fabricate the gold film-coated or gold wire-filled DC-PCF PBS which has the hexagonal arrangement of circular air holes.

In this paper, we propose a novel gold film-coated V-shape DC-PCF (V-DC-PCF) PBS based on the SPR effect. We compare the coupling lengths (CLs) of the X-polarization (X-pol) and Y-polarization (Y-pol) and the coupling length ratio (CLR) when the proposed V-DC-PCF PBS is coated with and without gold film. At the three wavelengths 1.610, 1.631, and 1.650 μm, the normalized output powers of the X-pol and Y-pol of the V-DC-PCF PBS are demonstrated when the propagation length increases. For the three splitting lengths (SLs) 188, 185, and 182 μm, the extinction ratio (ER) and insertion loss (IL) of the proposed V-DC-PCF PBS are investigated. Finally, we obtain a V-DC-PCF PBS with good performances, whose optimal SLs is 188 μm, ILs of the X-pol and Y-pol are less than 0.22 dB, and SB can cover the E + S + C + L + U band.

## 2. Design of the V-DC-PCF PBS

**Figure 1:**
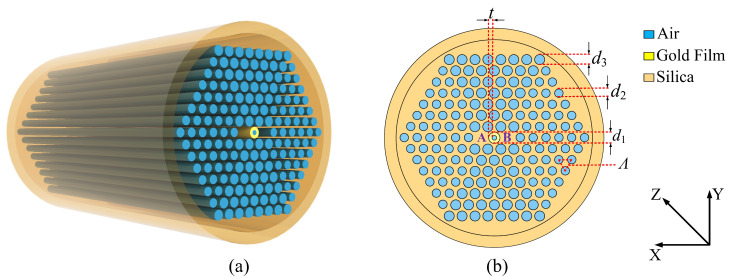
The three-dimensional (**a**) and cross-sectional (**b**) structures of the proposed V-shape dual-core photonic crystal fiber (V-DC-PCF) polarization beam splitter (PBS).

The three-dimensional and cross-sectional structures of the proposed V-DC-PCF PBS are shown in Figure 1a,b, respectively. From Figure 1a,b, the substrate material is silica, the air holes are arranged in a hexagonal lattice, and the hole to hole pitch is Λ. The most central air hole with the diameter of $d_{1}$ is coated with the gold film, which has a thickness of *t*. When the light energy is propagated inside the V-DC-PCF coated with gold film, the free electrons on the gold film surface interact with the incident light field, generating the SPR and exciting the surface plasmon polariton (SPP) mode on the gold film surface. At a specific wavelength, the core mode of the V-DC-PCF and SPP mode have the same propagation constant, so the mode coupling occurs due to the phase-matching condition. The two air holes in the first layer along the X-direction of the V-DC-PCF are missing to form the two cores, which are labeled as the cores A and B, respectively. In the cladding region of the V-DC-PCF, there are two other sizes of air holes. The diameter of the smaller air holes on the left and right sides is $d_{2}$, and the diameter of the larger air holes on the upper and lower sides is $d_{3}$. In practice, such a V-DC-PCF can be fabricated with the stack-and-draw method, and the gold film can be selectively coated on the most central air hole by the chemical vapor deposition or magnetron sputtering technique [33,40,47].

The material dispersion of the silica can be obtained by the Sellmeier equation as [49]

(1) $$n^{2}\left(\lambda \right)=1+\frac{A_{1}\lambda^{2}}{\lambda^{2}-B_{1}^{2}}+\frac{A_{2}\lambda^{2}}{\lambda^{2}-B_{2}^{2}}+\frac{A_{3}\lambda^{2}}{\lambda^{2}-B_{3}^{2}}$$

where λ is the wavelength of free space. The related parameters of the Sellmeier equation for the silica material are shown in Table 1.

The relative dielectric constant of the gold material can be described by the Drude-Lorentz model [50]

(2) $$\varepsilon_{m}=\varepsilon_{\infty }-\frac{\omega_{D}^{2}}{\omega (\omega -j\gamma_{D})}-\frac{\Delta \varepsilon \cdot \Omega_{L}^{2}}{\left(\omega^{2}-\Omega_{L}^{2}\right)-j\Gamma_{L}\omega }$$

where ω is the angle frequency of the guided-wave, $\varepsilon_{\infty }$ and Δε are the high frequency dielectric constant and weighted coefficient, $\omega_{D}$ and $\gamma_{D}$ are the plasma and damping frequencies, and $\Omega_{L}$ and $\Gamma_{L}$ are the frequency and bandwidth of the Lorentz oscillator, respectively. The specific parameters of the Drude-Lorentz model for the gold material are shown in Table 2.

The CLs in the X-pol and Y-pol directions of the V-DC-PCF PBS can be described as [51]

(3) $$CL_{X}=\frac{\lambda }{2\left|\left(n_{even}^{X}-n_{odd}^{X}\right)\right|}$$

(4) $$CL_{Y}=\frac{\lambda }{2\left|\left(n_{even}^{Y}-n_{odd}^{Y}\right)\right|}$$

where $CL_{X}$ and $CL_{Y}$ represent the CL of the X-pol and Y-pol, respectively, λ is the wavelength of the initial incident light, and $n_{even}^{X}$, $n_{odd}^{X}$, $n_{even}^{Y}$, and $n_{odd}^{Y}$ represent the effective refractive indices (ERIs) of the even and odd modes in the X-pol and Y-pol, respectively.

The CLR can be calculated by [52]

(5) $$CLR=\frac{CL_{Y}}{CL_{X}}$$

When the CLR = 2 or 1/2, the optimal SL can be obtained.

For the proposed V-DC-PCF PBS, we only need to consider the case that the initial incident light enters the core A or B since the geometric structure of the cores A and B are identical and symmetrical. In this work, when supposing that the initial incident light entering the core A, the output powers $P_{\mathrm{out}}$ of the X-pol and Y-pol in the core A can be described as [53]

(6) $$P_{\mathrm{out},A}^{X,Y}=P_{in}\cos^{2}\left(\frac{\pi }{2}\frac{PL}{CL_{X,Y}}\right)$$

where $P_{\mathrm{in}}$ is the power of the initial incident light, and PL is the propagation length inside the V-DC-PCF PBS.

The ER of the core A, which is considered as a significant parameter for evaluating the splitting performance of the V-DC-PCF PBS, can be calculated by [54]

(7) $$ER_{A}=10\mathrm{lo}g_{10}\frac{P_{out,A}^{X}}{P_{out,A}^{Y}}$$

The IL of the X-pol and Y-pol in the core A of the V-DC-PCF PBS can be described as [55]

(8) $$IL_{X,Y}=-10\mathrm{lo}g_{10}\frac{P_{out,A}^{X,Y}}{P_{in}^{}}$$

## 3. Simulation Results and Discussion

The finite element method is used to investigate the propagation characteristics of the proposed V-DC-PCF [56,57]. The initial fiber structure parameters are set as following: $d_{1}$ = 0.95 μm, $d_{2}$ = 1.20 μm, $d_{3}$ = 1.40 μm, Λ = 2.20 μm, and *t* = 55 nm. In the simulation, the material coefficients including the refractive indices of the silica and air and the relative dielectric constant of the gold material are set after the simulation model is established. Then, a perfect matching layer (PML), whose thickness is 10 μm and refractive index is $n_{\mathrm{silica}}$+0.03, is added to the outermost edge of the gold film-coated V-DC-PCF so as to absorb the radiation energy [58]. Moreover, the grid sizes of the silica, air holes, and PML are set as λ/4, and the grid size of the most central air hole coated with gold film is set as λ/6.

**Figure 2:**
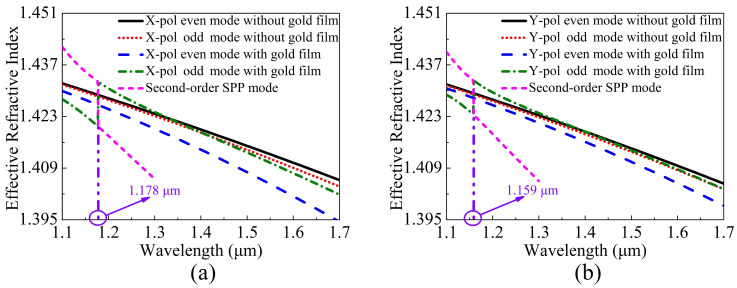
The ERIs of the (**a**) X-pol and (**b**) Y-pol even and odd modes and second-order surface plasmon polariton (SPP) mode calculated as functions of wavelength when the V-DC-PCF is coated without gold film and with gold film, respectively.

**Figure 3:**
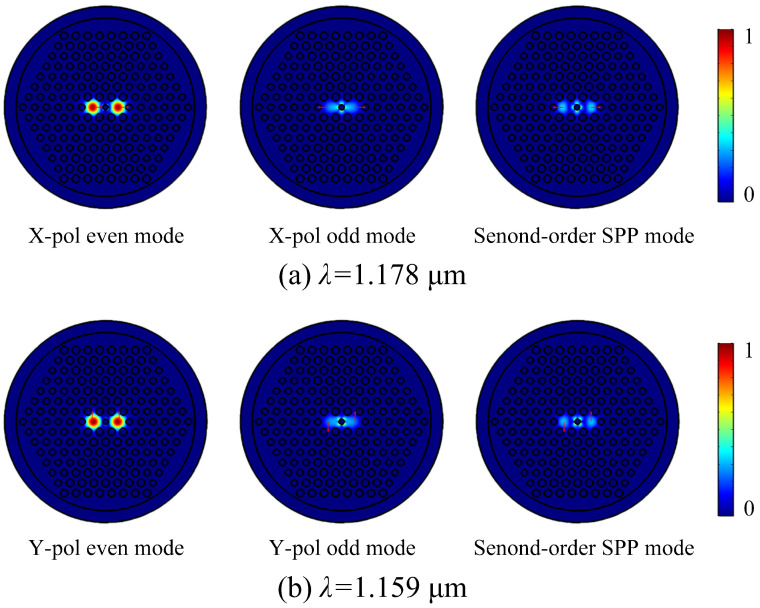
The mode field distributions of the (**a**) X-pol and (**b**) Y-pol even and odd modes and second-order SPP mode of the V-DC-PCF calculated at wavelengths 1.178 and 1.159 μm, respectively.

When the V-DC-PCF is coated without gold film and with gold film, the ERIs of the X-pol and Y-pol even and odd modes and second-order SPP mode are shown in Figure 2a,b, respectively. It can be seen from Figure 2a,b that for the V-DC-PCF without gold film, the ERIs of the X-pol and Y-pol even and odd modes decrease approximately linearly with the increase of wavelength. Moreover, the ERIs of the X-pol and Y-pol odd modes decrease more obviously than that of the X-pol and Y-pol even modes at the longer wavelength side, but the differences between the X-pol or Y-pol even and odd modes are very small. According to Equations (3) and (4), we can infer that the $CL_{X}$ and $CL_{Y}$ will also change in the approximately linear trend and have large values. In contrast, when the V-DC-PCF is coated with gold film, the ERIs of the X-pol and Y-pol even modes still decrease approximately linearly as the wavelength increases. However, there are two cross points between the ERIs of the X-pol and Y-pol odd modes and second-order SPP mode at wavelengths 1.178 and 1.159 μm, respectively, where the phase-matching condition is satisfied. According to the coupled mode theory, the X-pol and Y-pol odd modes occur to couple with the second-order SPP mode at wavelengths 1.178 and 1.159 μm, respectively. In addition, it can be seen from Figure 2a,b that before the phase-matching wavelengths, the ERIs of the X-pol and Y-pol odd modes decrease rapidly, along with a relatively small and stable slope. Hence, the differences between the ERIs of the X-pol and Y-pol even and odd modes increase rapidly with the increase of wavelength. The ERIs of the X-pol and Y-pol odd modes increase significantly at wavelengths 1.178 and 1.159 μm, respectively, and there are maximum differences between the ERIs of the X-pol and Y-pol even and odd modes. Thus, the $CL_{X}$ and $CL_{Y}$ will have significant changes at wavelengths 1.178 and 1.159 μm, respectively. But after the phase-matching wavelengths, the ERIs of the X-pol and Y-pol odd modes first decrease rapidly and then maintain a relatively stable slope. Finally, the relatively stable slope of the ERIs of the X-pol and Y-pol odd modes will be smaller than that of the ERIs of the X-pol and Y-pol even modes as the wavelength increases. Therefore, the differences between the ERIs of the X-pol and Y-pol even and odd modes first decrease and then increase as the wavelength increases. Figure 3a,b show the mode field distributions of the X-pol and Y-pol even and odd modes and second-order SPP mode calculated at wavelengths 1.178 and 1.159 μm, respectively. From Figure 3a,b, the mode field distributions of the X-pol and Y-pol even modes have no change at wavelengths 1.178 and 1.159 μm, respectively. But the mode field energies of the X-pol and Y-pol odd modes and second-order SPP mode occur to transfer at the two wavelengths. It further confirms the previous conclusion that only the X-pol and Y-pol odd modes and second-order SPP mode occur to couple at the phase-matching wavelengths.

**Figure 4:**
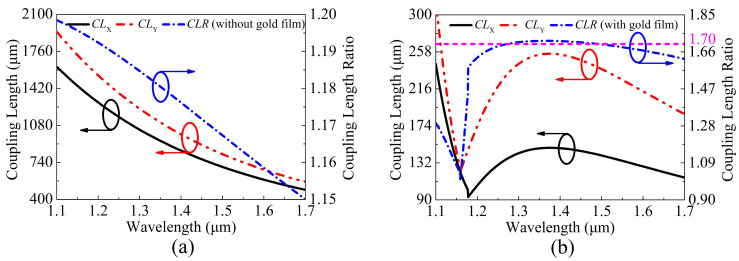
The $CL_{X}$, $CL_{Y}$, and CLR as functions of the wavelength when the V-DC-PCF is coated (**a**) without gold film and (**b**) with gold film.

The relationships between the $CL_{X}$, $CL_{Y}$, and CLR and wavelength are shown in Figure 4a,b, respectively, when the V-DC-PCF is coated without gold film and with gold film. It can be seen from Figure 4a that when the V-DC-PCF is coated without gold film, the $CL_{X}$ and $CL_{Y}$ decrease gradually, and the corresponding CLR also decreases in an approximately linear trend as the wavelength increases. According to Equations (3) and (4) and the above analysis, when the V-DC-PCF is coated with gold film, the $CL_{X}$ and $CL_{Y}$ decrease rapidly before the phase-matching wavelengths, and occur to change significantly at wavelengths 1.178 and 1.159 μm, respectively, as shown in Figure 4b. After the phase-matching wavelengths, the $CL_{X}$ and $CL_{Y}$ increase first and then decrease. At this time, according to Equation (5), the CLR decreases rapidly before wavelength 1.159 μm, has a significant change between wavelength 1.159 and 1.178 μm, and increases first and then decreases after wavelength 1.178 μm. However, after wavelength 1.178 μm, the overall change of the CLR is relatively flat. In addition, the CLR has the two intersections, where the CLR is equal to 1.7. By comparing Figure 4a,b, the maximum CL for the V-DC-PCF with gold film is smaller than the minimum CL for the V-DC-PCF without gold film, which has a direct effect on the SL of the V-DC-PCF PBS. From the above analysis, it is possible to achieve a PBS with the shorter SL and larger SB by using a V-DC-PCF with gold film.

**Figure 5:**
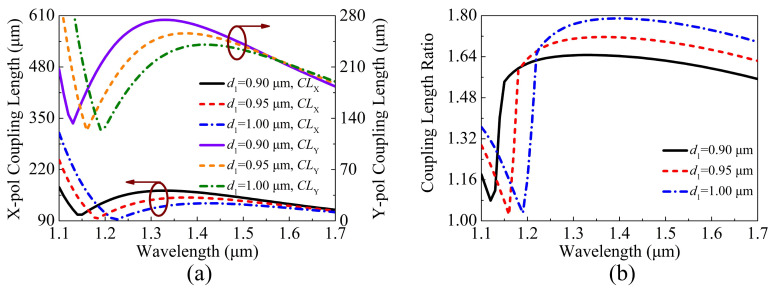
The variations of the (**a**) $CL_{X}$, $CL_{Y}$, and (**b**) CLR of the proposed V-DC-PCF PBS for different $d_{1}$.

The structure parameters of the proposed V-DC-PCF PBS with gold film need to be optimized to satisfy the condition of CLR = 2, which corresponds to the optimal SL. When the fiber structure parameters, including $d_{1}$, $d_{2}$, $d_{3}$, Λ, and *t* are changed, respectively, the ERIs of the X-pol and Y-pol even and odd modes will occur to change in different degrees, which can also cause the changes of the $CL_{X}$, $CL_{Y}$, and CLR. The variations of the $CL_{X}$, $CL_{Y}$, and CLR of the proposed V-DC-PCF PBS are shown in Figure 5a,b when $d_{1}$ is chosen as 0.90, 0.95, and 1.00 μm, respectively. It can be seen from Figure 5a that the $CL_{X}$ and $CL_{Y}$ decrease as $d_{1}$ increases at the short wavelength side. At the long wavelength side, as $d_{1}$ increases, the $CL_{X}$ still decreases slightly, while the $CL_{Y}$ shows a slightly increased trend. The CLR gradually increases as $d_{1}$ increases, and the phase-matching wavelength occurs to red-shift, as shown in Figure 5b.

**Figure 6:**
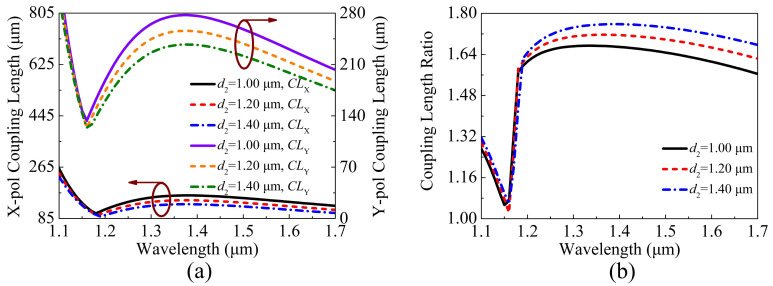
The variations of the (**a**) $CL_{X}$, $CL_{Y}$, and (**b**) CLR of the proposed V-DC-PCF PBS for different $d_{2}$.

The variations of the $CL_{X}$, $CL_{Y}$, and CLR of the proposed V-DC-PCF PBS are shown in Figure 6a,b when $d_{2}$ is chosen as 1.00, 1.20, and 1.40 μm, respectively. It can be seen from Figure 6a,b that as $d_{2}$ increases, the $CL_{X}$ and $CL_{Y}$ gradually decrease while the CLR shows an increased trend. In addition, the change of the phase-matching wavelength is not obvious, and only a slight red-shift occurs as $d_{2}$ increases.

**Figure 7:**
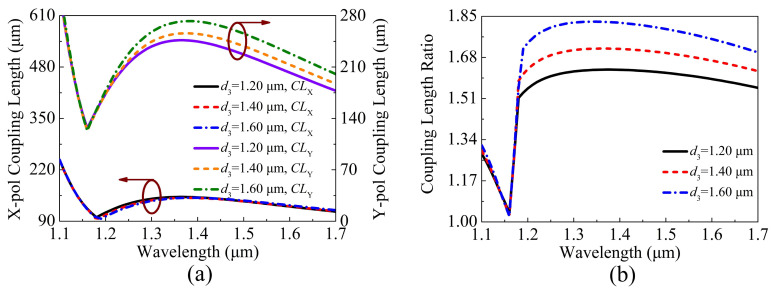
The variations of the (**a**) $CL_{X}$, $CL_{Y}$, and (**b**) CLR of the proposed V-DC-PCF PBS for different $d_{3}$.

When $d_{3}$ is chosen as 1.20, 1.40, and 1.60 μm, respectively, the variations of the $CL_{X}$, $CL_{Y}$, and CLR of the proposed V-DC-PCF PBS are shown in Figure 7a,b. From Figure 7a, as $d_{3}$ increases, the $CL_{X}$ decreases slightly at the short wavelength side and remains nearly unchanged at the long wavelength side. In comparison, the $CL_{Y}$ gradually increases as $d_{3}$ increases, and the corresponding change amplitude at the long wavelength side is larger than that at the short wavelength side. As shown in Figure 7b, as $d_{3}$ increases, the CLR gradually increases, and the position of the phase-matching wavelength remains unchanged.

**Figure 8:**
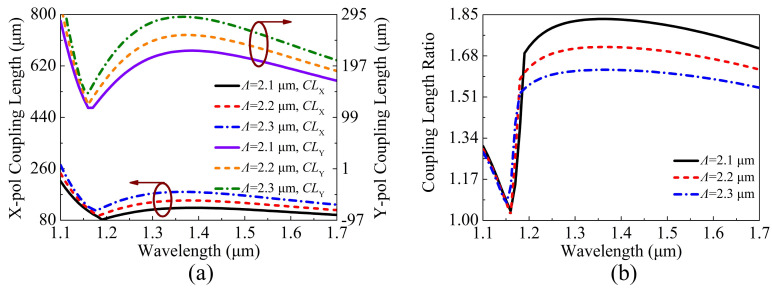
The variations of the (**a**) $CL_{X}$, $CL_{Y}$, and (**b**) CLR of the proposed V-DC-PCF PBS for different Λ.

The variations of the $CL_{X}$, $CL_{Y}$ and CLR of the proposed V-DC-PCF PBS are shown in Figure 8a,b when Λ is chosen as 2.1, 2.2, and 2.3 μm, respectively. Figure 8a,b, as Λ increases, the $CL_{X}$ and $CL_{Y}$ gradually increase, but the CLR shows a decreasing trend. In addition, the change of the phase-matching wavelength is not obvious, along with a small shift towards the short wavelength side. By comparing the results shown in Figure 5b, Figure 6b, Figure 7b, and Figure 8b, it is found that the change of Λ has the most remarkable influence on the CLR.

**Figure 9:**
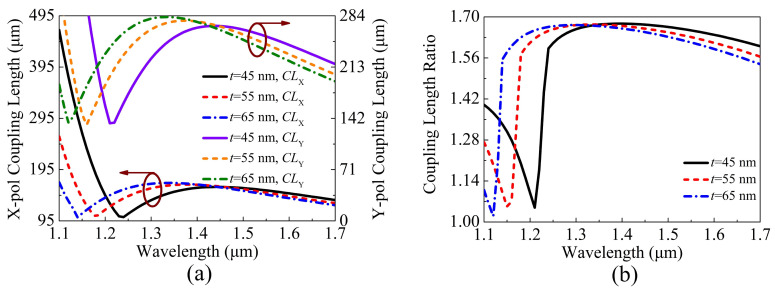
The variations of the (**a**) $CL_{X}$, $CL_{Y}$, and (**b**) CLR of the proposed V-DC-PCF PBS for different *t*.

Figure 9a,b show the variations of the $CL_{X}$, $CL_{Y}$, and CLR of the proposed V-DC-PCF PBS when *t* is chosen as 45, 55, and 65 nm, respectively. It can be seen from Figure 9a that as *t* increases, the $CL_{X}$ and $CL_{Y}$ increase at the short wavelength side and decrease at the long wavelength side. It can be seen from Figure 9b that as *t* increases, the CLR increases at the short wavelength side and decreases at the long wavelength side, and the position of the phase-matching wavelength gradually shifts towards the short wavelength side.

**Figure 10:**
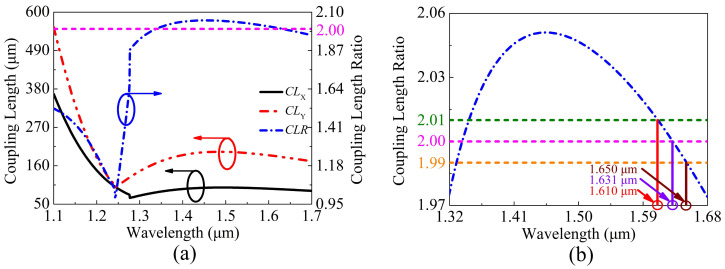
The optimal $CL_{X}$, $CL_{Y}$, and CLR of the proposed V-DC-PCF PBS, and (**b**) the zoomed flat region of the CLR.

Based on the above analysis, the influence rule of the structure parameters of the proposed V-DC-PCF PBS on the $CL_{X}$, $CL_{Y}$, and CLR can be clearly known. Thus, the empirical steps for designing such a V-DC-PCF PBS can be summarized as following. First, by together adjusting $d_{1}$ and *t*, the phase-matching condition can be achieved at the shorter wavelength, and the value of the CLR is close to 2. At this time, a relatively flat CLR curve can be obtained in the desired band. Second, by together adjusting $d_{2}$, $d_{3}$, and Λ, the CLR curve with the relatively flat profile and the value of 2 can be obtained when the phase-matching wavelength does not change obviously. Thus, the optimized structure parameters of the proposed V-DC-PCF PBS are chosen as follows: $d_{1}$ = 1.00 μm, $d_{2}$ = 1.41 μm, $d_{3}$ = 1.50 μm, Λ = 2.10 μm, and *t* = 46.5 nm. At this time, the variations of the optimal $CL_{X}$, $CL_{Y}$, and CLR of the proposed V-DC-PCF PBS and the corresponding zoomed flat region of the CLR are shown in Figure 10a,b, respectively. From Figure 10a, the $CL_{X}$ and $CL_{Y}$ have significant changes at the phase-matching wavelengths 1.277 and 1.243 μm, respectively. Moreover, the CLR is approximately equal to 2 in a wide wavelength range of above 1.277 μm. From Figure 10b, the value of the CLR changes from 1.974 to 2.051 in the wavelength range from 1.32 to 1.68 μm, and the maximum difference of the CLR between wavelength 1.32 and 1.68 μm is 0.077. In addition, the values of the CLR at wavelengths 1.610, 1.631, and 1.650 μm are 2.01, 2.00, and 1.99, respectively, which are approximately equal to 2. It is worth noting that although the value of the CLR is also equal to 1.99, 2.00, and 2.01 at other three shorter wavelengths, but the corresponding slope variation of the CLR is larger, which will affect the overall bandwidth to a great extent.

**Figure 11:**
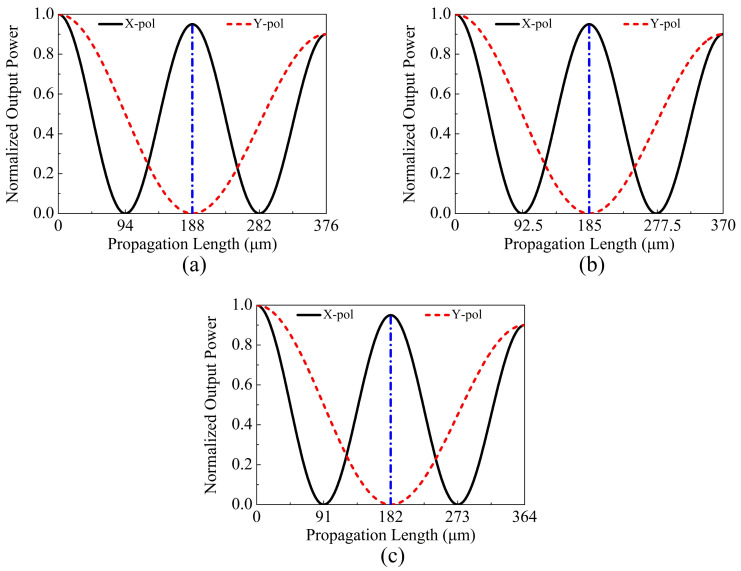
The relationships between the output powers $P_{out}$ of the X and Y-pol of the proposed V-DC-PCF PBS and PL at the three wavelengths (**a**) 1.610, (**b**) 1.631, and (**c**) 1.650 μm, respectively.

In the following, the relationships between the output powers $P_{out}$ of the X-pol and Y-pol of the proposed V-DC-PCF PBS and PL at the three wavelengths 1.610, 1.631, and 1.650 μm are shown in Figure 11a–c, respectively. From Figure 11a–c, $P_{out}$ of the X-pol reaches the maximum values when the PL is located at 188, 185, and 182 μm, respectively. In contrast, the corresponding $P_{out}$ of the Y-pol reaches 0 when the PL is located at 188, 185, and 182 μm, respectively. This phenomenon indicates that the X-pol light only exists in the core A while the Y-pol light only exists in the core B at the three PLs. Therefore, the SL of the proposed V-DC-PCF PBS may be 182, 185, or 188 μm. Moreover, another notable phenomenon is that the total $P_{out}$ decreases slightly as the PL increases. This is mainly because a fraction of the energy always propagates on the surface of the gold film, leading to the increase of the ohmic loss.

**Figure 12:**
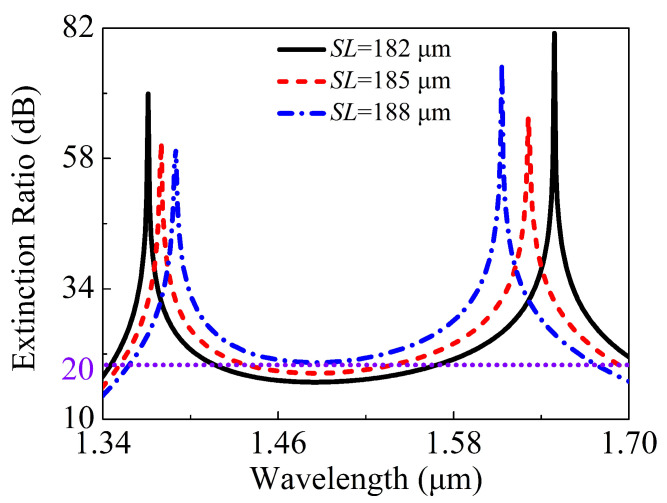
The ERs in the core A of the proposed V-DC-PCF PBS as functions of the wavelength for different SL.

**Figure 13:**
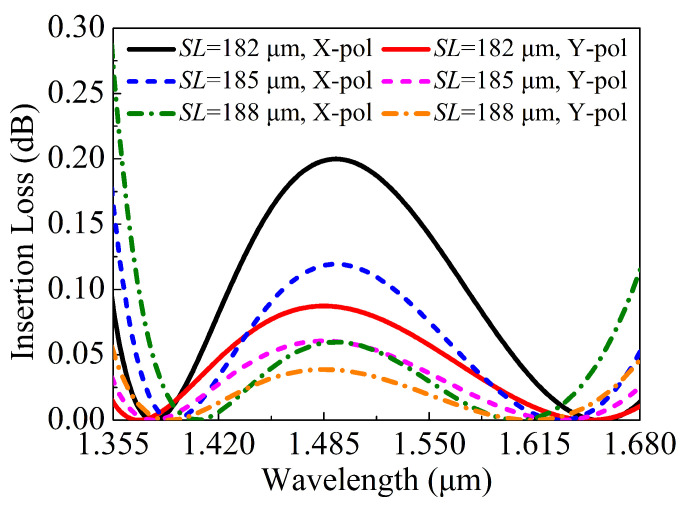
The ILs of the X-pol and Y-pol in the core A of the proposed V-DC-PCF PBS as functions of the wavelength for different SL.

When the SL is equal to 182, 185, and 188 μm, respectively, the relationships between the ERs in the core A of the proposed V-DC-PCF PBS and wavelength are shown in Figure 12. It can be seen from Figure 12 that for the SLs of 182 and 185 μm, the ERs are less than 20 dB in some wavelength ranges. In contrast, when SL is equal to 188 μm, the ER is always larger than 20 dB in a wide wavelength range from 1.359 to 1.677 μm. Figure 13 shows the relationships between the ILs of the X-pol and Y-pol in the core A of the proposed V-DC-PCF PBS and wavelength when the SL is equal to 182, 185, and 188 μm, respectively. From Figure 13, for the SLs of 182, 185, and 188 μm, the maximum IL of the X-pol and Y-pol is 0.22 dB in the wavelength range of 1.359 to 1.677 μm. Such a small IL can meet the actual application requirements. Because the proposed V-DC-PCF PBS has the ultra-short SL, the bending loss can be neglected. Therefore, we can draw a conclusion that for the proposed V-DC-PCF PBS, the optimal SL is 188 μm, the SB covers the entire E + S + C + L + U band, and the ILs of the X-pol and Y-pol are less than 0.22 dB.

The comparisons between the proposed V-DC-PCF PBS and reported SPR-based DC-PCF PBS are shown in Table 3. From Table 3, only the SB of the SPR-based DC-PCF PBS reported in Ref [12] is larger than that of the proposed V-DC-PCF PBS. However, in Ref [12], the SL and minimum IL are 4.036 mm and 0.8 dB, respectively, while they are only 188 μm and 0.22 dB in this work. Moreover, the air holes of the DC-PCF PBS reported in Ref [12] are arranged in a square lattice, so it is difficult to fabricate. In addition, only the SL of the SPR-based DC-PCF PBS reported in Ref [25] is shorter than that of this work. However, in Ref [25], the SB, which covers the S + C + L band, is much narrower than that of this work, and the IL is not given. Moreover, the air holes of square lattice of the DC-PCF PBS reported in Ref [25] also increase the difficulty of fabrication. In summary, the proposed V-DC-PCF PBS has the good comprehensive performances.

**Figure 14:**
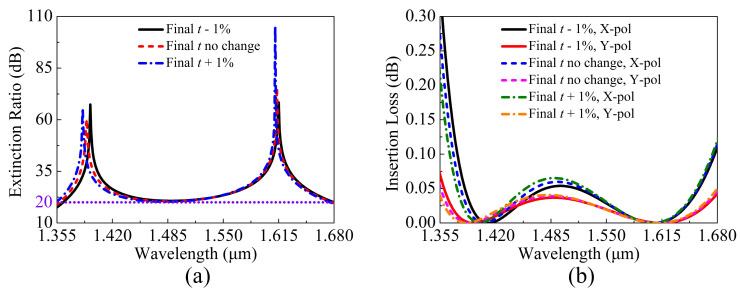
(**a**) ERs in the core A and (**b**) ILs of the X-pol and Y-pol in the core A of the proposed V-DC-PCF PBS as functions of wavelength when *t* has the distortion of ±1%.

The ERs in the core A and ILs of the X-pol and Y-pol in the core A of the proposed V-DC-PCF PBS are shown in Figure 14a,b when *t* changes ±1%. From Figure 14a, the wavelength range of the ER larger than 20 dB changes slightly, which indicates that the proposed V-DC-PCF PBS still has a wide SB. From Figure 14b, the ILs of the X-pol and Y-pol are always less than 0.22 dB in each SB. Thus, *t* has good error-tolerant rate in the actual coating process.

## 4. Conclusions

In summary, a novel gold film-coated V-DC-PCF PBS based on the SPR effect is proposed. The $CL_{X}$, $CL_{Y}$, and CLR of the proposed V-DC-PCF PBS coated without gold film and with gold film are investigated. The empirical steps for designing such a V-DC-PCF PBS are summarized by analyzing the effects of the fiber structure parameters on the $CL_{X}$, $CL_{Y}$, and CLR. The CLR of 2 can be obtained at the three wavelengths 1.610, 1.631, and 1.650 μm. When the SLs are equal to 188, 185, and 182 μm, the relationships between the ER, IL and wavelength are investigated, respectively. The proposed V-DC-PCF PBS has the good comprehensive performances, including the SL of 188 μm, IL of less than 0.22 dB, and SB of covering the E + S + C + L + U band. It is believed that the proposed V-DC-PCF PBS can find important applications in the laser, sensing, and dense wavelength division multiplexing systems.

## Figures and Tables

**Table 1:** The related parameters of the Sellmeier equation for the silica material.

$A_{1}$	$A_{2}$	$A_{3}$	$B_{1}\left(\mu m\right)$	$B_{2}\left(\mu m\right)$	$B_{3}\left(\mu m\right)$
0.6961663	0.4079426	0.8974794	0.0684043	0.1162414	9.896161

**Table 2:** The specific parameters of the Drude-Lorentz model for the gold material.

$\varepsilon_{\infty }$	Δε	$\omega_{D}/2\pi \left(\mathrm{THz}\right)$	$\gamma_{D}/2\pi \left(\mathrm{THz}\right)$	$\Omega_{L}/2\pi \left(\mathrm{THz}\right)$	$\Gamma_{L}/2\pi \left(\mathrm{THz}\right)$
5.9673	1.09	2113.6	15.92	650.07	104.86

**Table 3:** Comparisons between the proposed V-DC-PCF PBS and reported surface plasmon resonance (SPR)-based DC-PCF PBS.

Ref.	DC-PCF Structure	Gold Film or Wire	*SB*	*SL*	Max *IL*
[12]	Square lattice with circular air holes	Gold wire	O + E + S + C + L + U band	4.036 mm	0.8 dB
[13]	Hexagonal lattice with elliptical air holes	Gold wire	S + C + L + U band	254.6 μm	N/A
[14]	Hexagonal lattice with circular air holes	Gold wire	O and C bands	830 μm	N/A
[15]	D-shape hexagonal lattice with circular air holes	Gold film	C band	0.782 mm	1.5 dB
[16]	Hexagonal lattice with circular air holes	Gold wire	S + C + L + U band	577.5 μm	N/A
[17]	Rectangle lattice with circular air holes	Two gold wires	E + C band	1 mm	N/A
[18]	Hexagonal lattice with circular air holes	Gold film	S + C + L band	5.112 mm	N/A
[24]	Hexagonal lattice with circular air holes	Elliptical gold wire	C band	1.079 mm	N/A
[25]	Square lattice with circular air holes	Gold film	S + C + L band	47.26 μm	N/A
This work	Hexagonal lattice with circular air holes	Gold film	E + S + C + L + U band	188 μm	<0.22 dB
